# Characterizing cognitive function in patients with autoimmune encephalitis: an Australian prospective study

**DOI:** 10.1007/s00415-023-11967-w

**Published:** 2023-09-14

**Authors:** Sarah P. Griffith, Robb Wesselingh, Nabil Seery, Tiffany Rushen, Chris Kyndt, Brian Long, Udaya Seneviratne, Katherine Buzzard, Helmut Butzkueven, Terence J. O’Brien, Rubina Alpitsis, Charles B. Malpas, Mastura Monif, Australian Autoimmune Encephalitis Consortium

**Affiliations:** 1 Department of Neurosciences, Central Clinical School, Faculty of Medicine, Nursing and Health Sciences, Monash University, Level 6, Alfred Centre, 99 Commercial Road, Melbourne, VIC 3004 Australia; 2 Department of Neurology, Alfred Health, Level 6, Alfred Centre, 99 Commercial Road, Melbourne, VIC 3004 Australia; 3 Department of Neurology, Melbourne Health, 300 Grattan Street, Parkville, VIC 3050 Australia; 4 Department of Neurosciences, Eastern Health Clinical School, Box Hill Hospital, Monash University, Melbourne, VIC Australia; 5 Neuropsychology Unit, Monash Medical Centre, Monash Health, 246 Clayton Road, Clayton, VIC 3168 Australia; 6 Department of Neurosciences, Monash Health, Clayton Road, Clayton, VIC 3168 Australia; 7 Department of Medicine, Royal Melbourne Hospital, The University of Melbourne, Parkville, VIC 3052 Australia; 8 Melbourne School of Psychological Sciences, The University of Melbourne, Parkville, VIC 3052 Australia

**Keywords:** Autoimmune encephalitis, Cognitive outcomes, Neuropsychology, Autoimmune diseases

## Abstract

**Objective:**

This study uses the Wechsler intelligence and memory scales to characterize the cognitive function of patients with autoimmune encephalitis (AE) in the chronic stage of the disease. AE is a group of neuroinflammatory disorders, and cognitive impairment is a significant source of chronic morbidity in these patients.

**Methods:**

Fifty patients with an average disease duration of 3.2 years after diagnosis were prospectively recruited from four hospitals. They underwent a comprehensive cognitive examination using the Wechsler Abbreviated Scale of Intelligence (WASI-II), Wechsler Adult Intelligence Scale (WAIS-IV) and Wechsler Memory Scale (WMS-IV). Summary statistics were computed, and single-sample and independent-samples *t* tests were used to compare the cohort to normative data.

**Results:**

The results revealed significantly reduced performances in perceptual reasoning, processing speed, and working memory among AE patients. Seropositive AE patients exhibited below-norm processing speed, while the seronegative group showed reduced working memory and processing speed. Delayed memory performance was significantly below expectations only in seronegative patients. Pattern analysis indicated that intact cognition was the most observed outcome after AE, but significant heterogeneity was observed among the impaired patients.

**Conclusions:**

The study identified deficits in perceptual reasoning, processing speed, and working memory among chronic AE patients. Pattern analysis highlighted positive long-term cognitive outcomes for many but varied outcomes for those with ongoing difficulties. Although severely cognitively impaired patients were not included, the findings apply to  AE cohorts who attend outpatient clinical neuropsychology consultations emphasizing the need for thorough cognitive assessment. The results suggest a need for further research targeting other cognitive domains, including executive functions.

**Supplementary Information:**

The online version contains supplementary material available at 10.1007/s00415-023-11967-w.

## Introduction

Autoimmune encephalitis represents a group of non-infective neuroinflammatory disorders characterized by subacute cognitive changes with at least one of the following: new focal central nervous system (CNS) findings, seizures, cerebrospinal fluid (CSF) pleocytosis, and magnetic resonance imaging (MRI) features suggestive of encephalitis [1]. Patients in the acute (during admission), subacute (months) and chronic (years) phases post-initial diagnosis often exhibit cognitive dysfunction [2–9].

Studies examining autoimmune encephalitis (AE) have demonstrated that primary cognitive outcomes include memory impairments and some executive functions [2, 4]. The routine cognitive measures are largely heterogeneous, and thus far, it has been difficult to determine specific and differential cognitive profiles and deficits in different subsets of AE. Further, some centers do not conduct a specialist neurocognitive assessment of patients with AE due to limited resources or lack of expertise. In other centers, comprehensive testing may be available as part of research paradigms, but not for routine clinical care. These limitations preclude the generalizability of these findings to the clinical setting. Consequently, questions remain regarding the specific neuropsychological syndromes that may emerge after this disease.

Widely used scales of cognitive function, which are among the most robust assessment tools in routine clinical neuropsychology practice, have yet to be applied to the AE population. These scales have well-demonstrated reliability and validity, including reduced measurement error, well-documented discriminatory power, and a well-defined measurement model [10–12]. Applying these tools to a prospective population in a disease whose cognitive outcomes are not yet well understood may assist in establishing a foundational understanding of cognitive outcomes. Based on these findings, future studies of specific cognitive outcomes can then be derived.

In this study, we aim to prospectively characterize the cognitive profiles of AE patients at least 6 months after initial diagnosis by using several comprehensive scales of cognitive function, which examine verbal comprehension, perceptual reasoning, working memory, processing speed, auditory memory, visual memory, immediate memory and delayed memory indices.

## Methods

### Participants

Participants were recruited prospectively between October 2019 and April 2022. Patients were known to neurology clinics at four tertiary hospitals in Melbourne, Australia (Alfred Health, Monash Health, Eastern Health, and Melbourne Health) and recruited as part of the ongoing Australian Autoimmune Encephalitis Consortium study.

Included patients met the diagnostic criteria for possible autoimmune encephalitis as per Graus et al. position paper [1] and consented to participation in the study. Patients were also required to have no diagnosis or engagement in current investigations for a neurodegenerative illness (e.g., dementia of the Alzheimer’s type) and not experiencing a current major psychiatric episode (e.g., psychosis). None of the patients had a history of developmental language disorder or intellectual disability. Patients were classified into two groups: those with previously identified antibodies on CSF and/or serum (seropositive); and patients who met possible AE criteria but without an identifiable antibody (seronegative). Seropositive patients had antibody testing conducted as per their hospital’s corresponding procedures. For AE associated with specific antibodies, those antibodies had to be present in the CSF and/or serum with the highest sensitivity (e.g., anti-NMDAR ab-mediated AE present in CSF, anti-LGI1 ab-mediated AE antibodies present in serum).

#### Standard protocol approvals, registrations and patient consents

The central Human Research Ethics Committee at Alfred Health approved the study (HREC/17/Alfred/168). All participants, or their legally authorized representative, provided written consent to participate in the study.

### Assessment

All participants undertook a semi-structured clinical interview followed by formal psychometric testing. Sociodemographic variables (age, gender, education and employment) and clinical information were collected. Immunotherapy treatment data were collected, where the first-line immunotherapy was classified as IVIg and/or corticosteroids; second-line immunotherapy included rituximab or cyclophosphamide; and third-line immunotherapy included tocilizumab or bortezomib. Other variables were collected from medical records, when available, including ICU admission (y/n), modified Rankin scale (mRS) at discharge and number of anti-seizure medications (ASM) at the time of assessment. A qualified clinical neuropsychologist, SG, conducted all interviews and detailed cognitive testing.

The Wechsler Abbreviated Scale of Intelligence (WASI; 2nd edition) [12] was used to derive the indices of verbal comprehension (VCI; Vocabulary and Similarities subtests) and perceptual reasoning (PRI; Block Design and Matrix Reasoning subtests). The former measures word meanings and verbal abstract reasoning, with the latter reflecting fluid and visuospatial reasoning. The Wechsler Adult Intelligence Scale (WAIS; 4th edition) [13] was used to derive the processing speed index (PSI; Coding and Symbol Search subtests) and working memory index (WMI; Digit Span and Arithmetic subtests).

Wechsler Memory Scale (WMS; 4th edition) [11] Logical Memory, Visual Reproduction subtests, and the California Verbal Learning Test (CVLT 2nd edition) [14] learning and long-delay free recall equated scale scores were employed to derive the indices of auditory memory (AMI), visual memory (VMI), immediate memory (IMI), and delayed memory (DMI) as per the flexible approach, employing the ‘older adult’ configuration. This configuration allows for the substitution of Verbal Paired Associate I, and II scaled scores from the WMS-IV with the CVLT-II scores for the computation of the AMI, IMI, DMI [15].

The WASI-II, WAIS-IV and WMS-IV indices all have a mean of 100 and a standard deviation of 15 in the normative sample. The Australian language adapted forms were used where relevant. Data were collated into a Research Electronic Data Capture (REDCap) database.

### Analysis

Raw scores from cognitive tests were converted into standardized normed scores provided in the test manual. Per standard assessment and manual instructions, tests were then collated into the corresponding index and standardized to the data provided by Pearson assessments [11, 13]. An index was considered impaired at 1.5 standard deviations (SD) below the normative data (an index score of 78 or below). For sensitivity, graphs also demonstrate scores of 2.0 SD below the normative data.

Due to local COVID-19 restrictions during part of the recruitment process, five patients underwent a telehealth assessment. Hence, the processing speed and perceptual reasoning indexes could not be derived for these patients. Other reasons for incomplete indices included physical limitations (*N* = 2), too cognitively impaired for aspects of the battery (*N* = 3), and telehealth difficulties (*N* = 2).

Descriptive analysis of clinical variables and neuropsychology data was performed using summary statistics, where continuous and categorical variables were presented as mean (SD) and absolute number (percentage), respectively. A Chi-squared analysis with Yates correction was conducted to compare the frequency of outcomes observed to the frequency of expected outcomes for each test. Fisher’s exact tests were employed when Chi-squared analyses were invalid. Regression analysis investigated the relationship between time since symptom onset and cognitive outcome. Paired *t* tests were conducted separately to compare WASI/WAIS indices and WMS indices to investigate mean differences between the two indices. The frequency of psychometric patterns was determined through configural frequency analysis via the “*confreq*” package in R. This package computes the number of distant configurations of outcomes that could arise from the dataset. The package determines which configurations are present in the dataset and how many subjects match that configuration. Spearman’s correlation was used to investigate the relationship between indices and demographic and clinical variables. For binary and ordinal variables, Kendall’s tau was employed. Single-sample *t* tests determined the difference between normative data and AE cohorts (total, seropositive and seronegative). Independent-sample *t* tests were used to determine the difference between the means of the AE cohorts (including total, seropositive, and seronegative). Missing data were handled via pairwise deletion. Using Levene’s test (variance) and Shapiro–Wilk (normality), statistical assumptions were checked. If non-normal, nonparametric test (Mann–Whitney *U* test) was used. All data were analyzed using JASP (version 0.14.1) and R Studio (version 2022.02.3) with R (version 4.2.1) with the following packages installed: “*confreq*”.

## Results

### Patient characteristics

Fifty people with possible autoimmune encephalitis were recruited prospectively into the study. The cohort comprised 26 females and 24 males (see Table 1). Forty-eight percent of the cohort had a diagnosis of seropositive AE. Of these, 10 with anti-NMDAR ab-mediated AE, 9 with anti-leucine-rich glioma-inactivated-1 (LGI-1) ab-mediated AE, 2 with contactin-associated protein-like 2 (CASPR-2) ab-mediated AE, and 1 with voltage-gated potassium channel complex (unspecified; VGKC) ab-mediated AE antibodies, and 2 with other antibodies. The average time between symptom onset and neuropsychological testing was 3.28 years.

**Table 1:** Key demographic data

Characteristics	Mean (SD) [range]	*N*
Age	52.04 (18.48) [21.00–81.00]	50
Sex, male [*N* (%)]	26 (52)	50
Months between symptom onset and neuropsychological assessment	40.10 (32.62) [6.00, 126]	49
Months between hospital admission and neuropsychological assessment	37.75 (31.24) [3, 121]	49
Months between symptom onset to hospital admission	2.30 (5.27) [0, 30]	49
Education, years	12.96 (2.79) [7–18 years]	50
Telehealth [*N* (%)]	5 (10.00)	50
Seropositive [*N* (%)]	24 (48.00)	50
Anti-NMDAR ab-mediated AE	10 (20.83)	24
Anti-LGI1 ab-mediated AE	9 (18.75)	24
Anti-CASPR2 ab-mediated AE	2 (4.17)	24
Anti-VGKC ab-mediated AE	1 (2.08)	24
Other antibodies	2 (4.17)	24
Seronegative [*N* (%)]	26 (52.00%)	24
ASM use [*N* (%)]		
0 ASM	24 (50.00)	48
1 ASM	11 (22.92)	48
2 + ASM	13(27.08)	48
Treatment line [*N* (%)]		
First line	49 (100.00)	50
Second line	27 (55.10)	50
Third line	0 (100.00)	50
mRS at discharge [*N* (%)]		
1	9 (19.56)	46
2	18 (39.13)	46
3	16 (34.78)	46
4	3 (6.522)	46
ICU Admission during main hospital admission [*N* (%)]	22 (47.83)	46

*ASM* anti-seizure medication, *ICU* intensive care unit, *mRS* modified Rankin scale

To compare differences across clinic and demographic variables, independent-sample *t* tests were employed. There was a significant difference between sex and age, Mann–Whitney *U* (*N*_Female_ = 24, *N*_Male_ = 26) = 202.00, *p* = 0.033. There were no other significant differences.

### Verbal comprehension, perceptual reasoning, processing speed and working memory characteristics

**Fig. 1:**
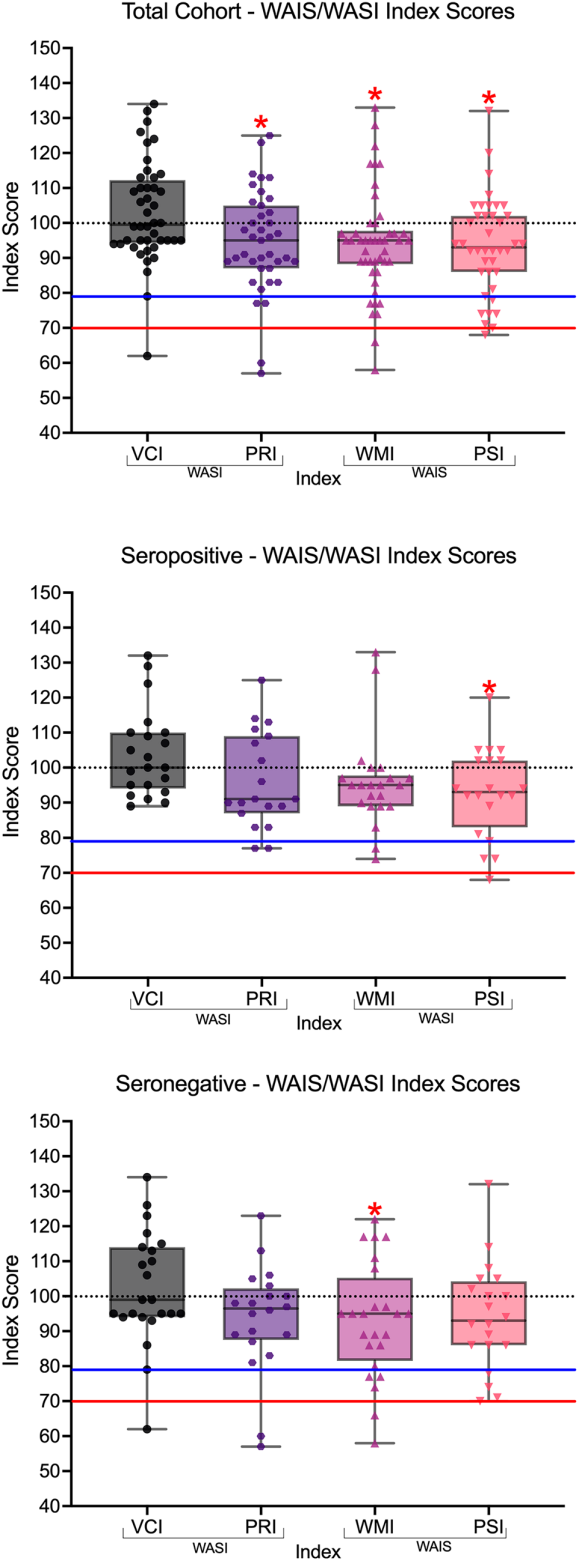
Box plot of participant’s scores on the WAIS-IV (Wechsler Adult Intelligence Scale, 4th edition) and WASI-II (Wechsler Abbreviated Scale of Intelligence, 2nd edition) indices. The normative data mean is denoted by the black dotted line. Scores below the blue line are 1.5 standard deviations below the normative mean (index score of 78 or below) and are considered mildly impaired. Scores below the red line are 2.0 standard deviations below the normative mean (index score of 80 of below) and are considered severely impaired. *VCI* verbal comprehension index, *PRI* perceptual reasoning index, *WMI* working memory index, *PSI* processing speed index

WAIS, WASI, and WMI index scores by total cohort, seropositive and seronegative, are presented in Table 2. Figure 1 provides a visualization of the distribution of the scores for the total cohort, seropositive cohort and seronegative groups. Subtest descriptive data are available in the supplementary data, Table S1.

**Table 2:** Index and subtests characteristics of the entire cohort, seropositive and seronegative

Index	Entire cohort				
	*N*	*M* (SD)	Min	Max	% Impaired at 1.5 SD below normative mean
Verbal comprehension (VCI)	44	103.00 (14.37)	62	134	2.27
Perceptual reasoning (PRI)	39	94.72 (14.53)	57	125	10.26
Working memory (WMI)	46	94.26 (14.97)	58	133	15.21
Processing speed (PSI)	40	93.43 (13.91)	68	132	17.50
Auditory memory (AMI)	43	95.74 16.58)	49	123	16.28
Visual memory (VMI)	43	98.95 (21.17)	45	137	18.60
Immediate memory (IMI)	43	97.28 (14.70)	51	128	6.98
Delayed memory (DMI)	43	95.53 (20.50)	43	133	16.28

	Seropositive				
	*N*	*M* (SD)			
Verbal comprehension (VCI)	21	104.00 (12.49)	89	132	0.00
Perceptual reasoning (PRI)	19	96.00 (13.67)	77	125	10.53
Working memory (WMI)	22	95.64 (13.26)	74	133	9.09
Processing speed (PSI)	20	92.80 (12.80)	68	120	15.00
Auditory memory (AMI)	21	99.67 (13.99)	62	123	4.76
Visual memory (VMI)	21	102.10 (20.43)	63	137	14.29
Immediate memory (IMI)	21	100.10 (14.40)	79	128	0.00
Delayed memory (DMI)	21	101.50 (17.44)	67	133	9.52

	Seronegative				
	*N*	*M* (SD)			
Verbal comprehension (VCI)	23	102.10 (16.12)	62	134	4.34
Perceptual reasoning (PRI)	20	93.50 (15.54)	57	123	10.00
Working memory (WMI)	24	93.00 (16.57)	58	122	20.83
Processing speed (PSI)	20	94.05 (15.24)	70	132	20.00
Auditory memory (AMI)	22	92.00 (18.25)	49	120	27.27
Visual memory (VMI)	22	95.91 (21.89)	45	137	22.72
Immediate memory (IMI)	22	94.55 (14.78)	51	118	13.63
Delayed memory (DMI)	22	89.82 (21.93)	43	120	22.27

Regression analyses investigating the relationship between time since symptom onset and recruitment, and cognitive outcome were non-significant (see Supplementary Material, Figure S1).

Single-sample *t* tests demonstrated that for the whole cohort, the PRI, WMI and PSI were significantly below the normative mean (Table 3). In the seropositive cohort, the PSI was significantly below the normative mean. In the seronegative cohort, the WMI and the PSI were significantly below the normative mean (Table 3).

**Table 3:** Cohort differences from WAIS/WASI normative data

Cohort	*t*	*df*	*p*	Cohen’s *d*	95% CI of Cohen’s *d*	
	Total cohort				Lower	Upper
Verbal comprehension (VCI)	1.39	43.00	0.17	0.21	− 0.09	0.51
Perceptual reasoning (PRI)	− 2.27	38.00	0.03*	− 0.36	− 0.69	− 0.04
Working memory (WMI)	− 2.60	45.00	0.01*	− 0.38	− 0.68	− 0.08
Processing speed (PSI)	− 2.99	39.00	0.005*	− 0.47	− 0.80	− 0.14

	Seropositive					
Verbal comprehension (VCI)	1.45	20.00	0.16	0.32	− 0.13	0.75
Perceptual reasoning (PRI)	− 1.28	18.00	0.28	− 0.29	− 0.75	0.17
Working memory (WMI)	− 1.54	21.00	0.14	− 0.33	− 0.76	0.10
Processing speed (PSI)	− 2.52	19.00	0.02*	− 0.56	− 1.03	− 0.08

	Seronegative					
Verbal comprehension (VCI)	0.63	22.00	0.53	0.13	− 0.28	0.54
Perceptual reasoning (PRI)	− 1.87	19.00	0.08	− 0.42	− 0.87	0.05
Working memory (WMI)	− 2.07	23.00	0.05*	− 0.42	− 0.84	0.00
Processing speed (PSI)	− 1.75	19.00	0.01*	− 0.39	− 0.84	0.07

For Student’s *t* test, the alternative hypothesis specifies that the mean is different from 100

*Significant at *p* ≤ 0.05

### Paired *t*-tests between WAIS/WASI indices

Paired-samples *t* tests revealed significant differences between VCI (*M* = 103.00, SD = 14.37) and PRI(*M* = 94.72, SD = 14.53, *t*(37) = 2.40, *p* = 0.02), VCI and VMI (*M* = 94.26, SD = 14.97, *t*(43) = 3.40, *p* = 0.001), VCI and PSI (*M* = 93.43, SD = 13.91, *t*(37) = 2.82, *p* = 0.008). There were no significant differences between PRI, and PSI, PRI and WMI, and WMI and PSI (Table S2).

### Correlations between demographic and clinical variables and the WAIS/WASI indices

There was a statistically significant negative correlation between PRI and the number of ASMs (*τ*_b_ = − 0.35, *p* = 0.008). For PSI, there were significantly negative correlations between the time between main hospital admission and assessment (*r*(37) = − 0.32, *p* = 0.046) and time between symptom onset and assessment (*r*(37) = − 0.32, *p* = 0.047).

### Comparison of different sub-types of AE in the WAIS/WASI

*Seropositive versus seronegative AE* When patients with and without antibody status were compared, there was no significant difference across all the indices (Supplementary Data; Table S3).

*Anti-NMDAR versus anti-LGI1 ab-mediated AE* When patients with anti-NMDAR ab-mediated AE were compared to those with anti-LGI1, there was no significant difference across all the indices (Supplementary Data; Table S4).

*Anti-NMDAR versus all other seropositive AE patients* When patients with anti-NMDAR ab-mediated AE were compared to the rest of the seropositive cohort, there was no significant difference across all the indices (Supplementary Data; Table S5).

*Anti-LGI-1 antibody mediated AE versus all other seropositive AE patients* When patients with anti-LGI1 antibodies were compared to the rest of the seropositive cohort, there was no significant differences across all the indices (Supplementary Data; Table S6).

### Frequency of impairments in WAIS (Wechsler Adult Intelligence Scale)/WASI (Wechsler Abbreviated Scale of Intelligence)

When a deficit was defined at 1.5 SD below the normative mean, PSI deficit in the entire AE patient cohort was the most frequent at 17.50% (95% CI 7.34, 32.78). This was followed by deficits in WMI at 15.22% (95% CI 6.34, 28.87), PRI at 10.26% (95% CI 2.80, 24.22) and VCI at 2.57% (95% CI 0.00, 12.02). For the seropositive cohort, again, PSI was the most common deficit at 15.00% (95% CI 3.21, 37.89), followed by PRI at 10.53% (95% CI 1.30, 33.14), WMI at 9.09% (95% CI 1.12, 29.16) and VCI with 0% (95% CI 0.00, 16.11). For the seronegative cohort, WMI was the most frequent deficit at 20.83% (95% CI 7.13, 42.15), followed by PSI at 20.00% (95% CI 5.73, 43.66), then PRI at 10.00% (95% CI 1.24, 31.7) and VCI at 4.35% (95% CI 0.11, 21.95).

In the total cohort, when impairment was defined as 1.5 SD below the normative mean, Chi-square analysis demonstrated no significant differences between frequency of expected performances and frequency of actual performance for the VCI [*χ*^2^(1, *N* = 44) = 0.26, *p* = 0.61], PRI [*χ*^2^(1, *N* = 39) = 0, *p* = 1], WMI [*χ*^2^(1, *N* = 46) = 1.01, *p* = 0.31] and PSI [*χ*^2^(1, *N* = 40) = 1.03, *p* = 0.31]. In the seropositive cohort, the Chi-square analysis violated statistical assumptions for the VCI. No significant differences were observed for the PRI [*χ*^2^(1, *N* = 19) = 0, *p* = 1], WMI [*χ*^2^(1, *N* = 22) = 0.28, *p* = 0.60], and PSI [*χ*^2^(1, *N* = 20) = 0.28, *p* = 0.60]. In the seronegative cohort, no significant differences were found for the VCI [*χ*^2^(1, *N* = 23) = 0.48, *p* = 0.49], PRI [*χ*^2^(1, *N* = 20) = 0.00, *p* = 1.00], WMI [*χ*^2^(1, *N* = 24) = 0.78, *p* = 0.38], and PSI [*χ*^2^(1, *N* = 20) = 0.91, *p* = 0.34].

When a deficit was defined at 2.0 SD below the normative mean, in the total cohort, the most frequent deficit was in the PRI at 5.13% (95% CI 0.63, 17.32). This was followed by PSI at 5.00% (95% CI 0.61, 16.92), WMI at 4.35 (95% CI 0.53, 14.84) and VCI at 2.27 (95% CI 0.00, 12.02). For the seropositive cohort, PSI was the most common deficit at 5.00% (95% CI 0.13, 24.87). VCI, PRI and WMI all were 0% (95% CI VCI 0.00, 16.11; PRI 0.00, 17.65; WMI 0.00, 15.44) for the seropositive group with the deficit being defined at 2.0 SD below the normative mean. For the seronegative cohort, PRI was the most common deficit at 10% (95% CI 1.24, 31.70), followed by WMI at 8.33% (95% CI 1.03, 27.00), PSI at 5.00% (95% CI 0.13, 24.87) and VCI at 4.50% (95% CI 0.11, 21.95).

For the total cohort, no significant differences between frequency of observed performance and frequency of expected performance were noted for the VCI [*χ*^2^(1, *N* = 44) = 0.51, *p* = 0.47], PRI [*χ*^2^(1, *N* = 39) = 0, *p* = 1], WMI [*χ*^2^(1, *N* = 46) = 0, *p* = 1] and PSI [*χ*^2^ (1, *N* = 40) = 0, *p* = 1]. For the seropositive cohort, Fisher’s exact tests did not indicate a significant association between expected and observed values for VCI, PRI, WMI and PSI (*p* = 1). For the seronegative cohort, there were no significant differences from frequency of expected performances for the VCI [*χ*^2^(1, *N* = 23) = 0.522 *p* = 0.47], PRI [*χ*^2^(1, *N* = 20) = 0.00, *p* = 1.00], WMI [*χ*^2^(1, *N* = 24) = 0, *p* = 1] and PSI [*χ*^2^(1, *N* = 20) = 0.53, *p* = 0.47].

### Wechsler Memory Scale (WMS) in AE

**Fig. 2:**
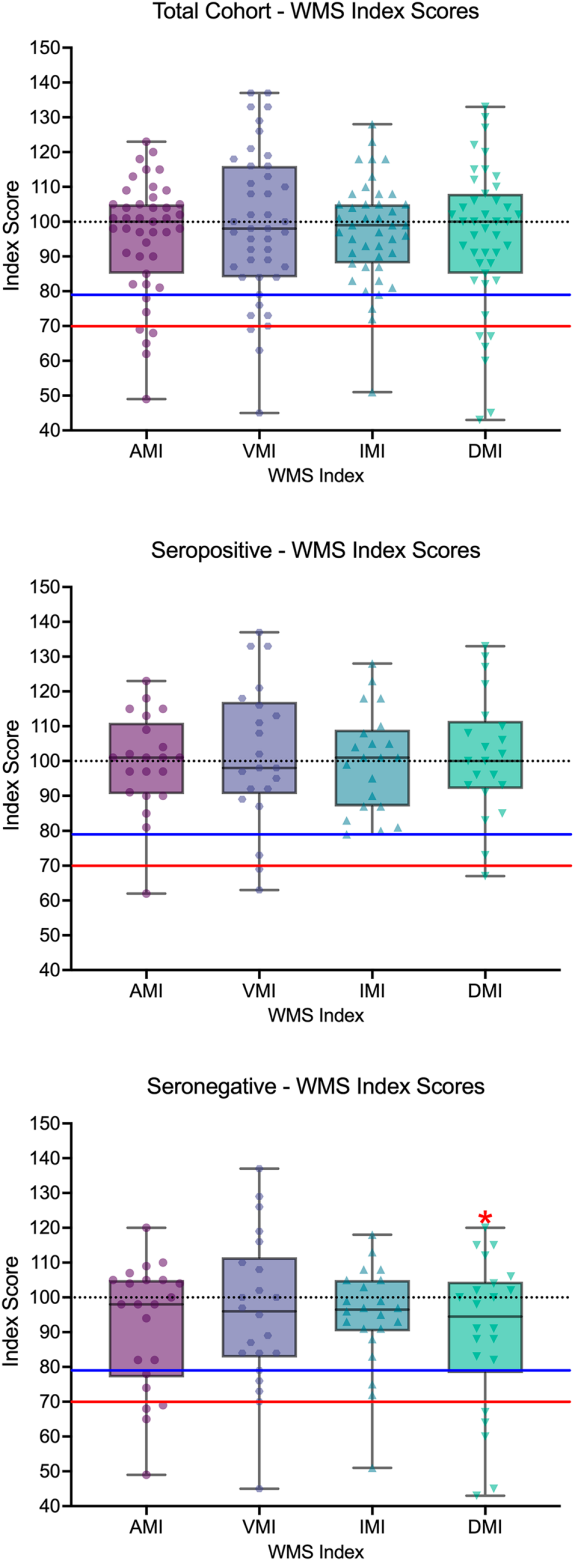
Box plot of participant’s scores on the WMS indices. The normative data mean is denoted by the black dotted line. Scores below the blue line are 1.5 standard deviations below the normative mean (index score of 78 or below). Scores below the red line are 2.0 standard deviations below the normative mean (index score of 80 of below). *AMI* auditory memory index, *VMI* visual memory index, *IMI* immediate memory index, *DMI* delayed memory index. *Significant at *p* ≤ 0.05

WMS Index scores by total cohort, seropositive and seronegative are presented in Table 2. Figure 2 illustrates the distribution of scores. Single-sample *t* tests demonstrated that in the seronegative cohort, the DMI was significantly below the normative mean (Table 4). Subtest descriptive data are available in the Supplementary Data, Table S1.

**Table 4:** Difference in Wechsler Memory Scale (WMS) between AE patients and normative data

Cohort	*t*	*df*	*p*	Cohen’s *d*	95% CI of Cohens *d*	
	Total cohort				Lower	Upper
Auditory memory (AMI)	− 1.68	42.00	0.10	− 0.26	− 0.56	0.05
Visual memory (VMI)	− 0.32	42.00	0.75	− 0.05	− 0.35	0.25
Immediate memory (IMI)	− 1.21	42.00	0.23	− 0.19	− 0.49	0.12
Delayed memory (DMI)	− 1.43	42.00	0.16	− 0.22	− 0.52	0.09

	Seropositive					
Auditory memory (AMI)	− 0.11	20.00	0.91	− 0.02	− 0.45	0.40
Visual memory (VMI)	0.48	20.00	0.64	0.11	− 0.33	0.53
Immediate memory (IMI)	0.05	20.00	0.96	0.01	− 0.42	0.44
Delayed memory (DMI)	0.40	20.00	0.69	0.09	− 0.34	0.52

	Seronegative					
Auditory memory (AMI)	− 2.06	21.00	0.05	− 0.44	− 0.87	0.01
Visual memory (VMI)	− 0.88	21.00	0.39	− 0.19	− 0.61	0.24
Immediate memory (IMI)	− 1.73	21.00	0.10	− 0.37	− 0.80	0.07
Delayed memory (DMI)	− 2.18	21.00	0.041*	− 0.46	− 0.90	− 0.02

For single-sample *t* tests, the null hypothesis was that the population mean is 100

*Significant at *p* ≤ 0.05

### Paired *t*-tests between WMS indices

There were no significant differences between AMI and VMI, AMI and IMI, AMI and DMI, VMI and IMI, VMI and DMI and IMI and DMI (Table S2).

### Correlations between demographic and clinical variables and the WMS indices

There was a significant negative correlation between the IMI and the number of ASM (*τ*_b_ = − 0.314, *p* = 0.013). There were significant correlations between sex and the VMI, IMI and DMI indices [(*τ*_b_ = 0.37, *p* = 0.003; *τ*_b_ = 0.29, *p* = 0.023; *τ*_b_ = 0.31, *p* = 0.015), respectively], which suggested that men scored higher than females. MRS at discharge was negatively associated with the AMI score (*τ*_b_ = − 0.29, *p* = 0.002).

### Comparison of different sub-types of AE in the Wechsler Memory Scale (WMS)

*Seropositive vs. seronegative AE* When patients with and without antibody status were compared, there was no significant difference in WMS in the two groups (Supplementary Data; Table S3).

*Anti-NMDAR ab-mediated AE versus anti-LGI1 ab-mediated AE* When patients with anti-NMDAR ab-mediated AE were compared to those with anti-LGI1, there were no significant differences across all indices of WMS (Supplementary Data; Table S4).

*Anti-NMDAR ab-mediated versus all other seropositive AE patients* When patients with anti-NMDAR ab-mediated AE were compared to the rest of the seropositive cohort, there was no significant difference in WMS between the two groups (Supplementary Data; Table S5).

*Anti-LGI-1 ab-mediated AE versus all other seropositive AE patients* When patients with anti-LGI1 ab-mediated AE were compared to the rest of the seropositive cohort, there was no significant difference in WMS indices between the two groups (Supplementary Data; Table S6).

### Frequency of impairments in WMS (Wechsler Memory Scale)

When a deficit was defined at 1.5 SD below the normative mean, in the total cohort, the most frequent deficit was in the DMI at 18.60% (95% CI 6.81, 30.70) followed by IMI at 17.50% (1.46, 19.06), and VMI and AMI at 16.28% (95% CI 8.39, 33.40 and 6.81, 30.70, respectively). In the seropositive cohort, the most common deficit was the VMI at 14.29% (95% CI 3.05, 36.34) followed by DMI at 9.52% (1.18, 30.38), AMI at 4.76% (0.12, 23.82) and IMI at 0% (95% CI 0.00, 16.11). In the seronegative cohort, AMI was the most common deficit at 27.27% (95% CI 10.73, 50.22), followed by VMI at 22.73% (95% CI 7.82, 45.37), DMI at 22.73% (95% CI 9.82, 45.37) and IMI at 13.64% (95% CI 2.91, 34.91).

In the total cohort, when impairment was defined as 1.5 SD below the normative mean, Chi-square analysis examining differences between frequency of actual performance and observed performance showed no significant differences for the AMI [*χ*^2^(1, *N* = 43) = 1.02, *p* = 0.31], VMI [*χ*^2^(1, *N* = 43) = 1.67, *p* = 0.20], IMI [*χ*^2^(1, *N* = 43) = 0.18, *p* = 0.67] and DMI [*χ*^2^(1, *N* = 43) = 1.02, *p* = 0.31]. For the seropositive cohort, the IMI violated statistical assumptions. There were no significant differences observed for the AMI [*χ*^2^(1, *N* = 21*)* = 0.53, *p* = 0.47], VMI [*χ*^2^(1,* N* = 21) = 0.28, *p* = 0.60] and DMI [*χ*^2^(1,* N* = 21) = 0, *p* = 1]. For the seronegative cohort, there was no significant difference between frequency of expected performance and frequency of observed performance reported for the AMI [*χ*^2^(1, *N* = 22) = 1.38, *p* = 0.24], VMI [*χ*^2^(1, *N* = 22) = 0.68, *p* = 0.41], IMI [*χ*^2^(1, *N* = 22) = 0, *p* = 1] and DMI [*χ*^2^(1, *N* = 22) = 0.68, *p* = 0.41].

When a deficit was defined at 2.0 SD below the normative mean, in the total cohort, the most common deficit was the DMI at 13.95% (95% CI 5.30, 27.93), followed by AMI at 11.63% (95% CI 3.8, 25.08), VMI at 9.30% (95% CI 2.59, 22.14) and IMI at 2.33% (95% CI 0.06, 12.29). In the seropositive cohort, the most common deficit was VMI at 9.52% (95% CI 1.18, 30.38), DMI and AMI were both 4.76% (95% CI 0.12, 32.82 and 0.12, 23.82, respectively), and IMI was 0% (95% CI 0.00, 16.11). In the seronegative cohort, DMI was the most common deficit at 22.73% (95% CI 7.82, 45.37), followed by AMI at 18.18% (95% CI 5.19, 40.28), VMI at 9.09% (95% CI 1.12, 29.16) and IMI at 4.55% (95% CI 0.12, 22.84).

For the total cohort, when impairment was defined at 2.0 SD below the normative mean, no significant differences between frequency of expected performances and frequency of observed performances were noted for the AMI [*χ*^2^(1, *N* = 43) = 1.61 *p* = 0.20], VMI [*χ*^2^(1,43) = 0.85 *p* = 0.36], IMI [*χ*^2^(1,43) = 0.51, *p* = 0.47] and DMI [*χ*^2^(1,43) = 2.49, *p* = 0.11]. For the seropositive cohort, Fisher’s exact tests for the AMI, VMI, IMI and DMI did not indicate significant association between observed and expected results (*p* = 1, *p* = 0.48, *p* = 1 and *p* = 1, respectively). For the seronegative cohort, no significant differences were found for the AMI [*χ*^2^(1, *N* = 22) = 0.90, *p* = 0.34], VMI [*χ*^2^(1, *N* = 22) = 0, *p* = 1], IMI [*χ*^2^(1, *N* = 22) = 0.52, *p* = 0.47] and DMI [*χ*^2^(1, *N* = 22) = 1.74, *p* = 0.18].

### Patterns of cognitive impairment in AE

Using pattern analysis, of the 256 possible patterns that could emerge from the data, 11 patterns were observed when including only patients who could complete the entire cognitive batteries (Table 5). This initial assessment excluded patients (*N* = 4) who were unable to complete the battery due to cognitive reasons (i.e., having severe enough deficits that limited their capacity to participate or complete the test). The subsequent analysis included the above 4 patients, and once again, of the 256 possible patterns that could emerge from the data, 11 were observed, with five patients now meeting global impairment.

**Table 5:** Patterns of cognitive impairments observed in the AE cohort

Patterns of psychometric impairment	*N* (%)
Intact	22 (59.46)
Visual memory	3 (8.11)
Auditory memory	1 (2.7)
Auditory memory and delayed memory	2 (5.4)
Processing speed	3 (8.11)
Working memory, visual memory and delayed memory	1 (2.7)
Working memory, auditory memory and delayed memory	1 (2.7)
Working memory, auditory memory and immediate memory	1 (2.7)
Perceptual reasoning, processing speed, visual memory and delayed memory	1 (2.7)
Perceptual reasoning, working memory, visual memory	1 (2.7)
Global impairment	1 (2.7)
Total	37

## Discussion

Autoimmune encephalitis-related cognitive impairment is a major source of morbidity affecting many aspects of the patient’s life [16]. Establishing a comprehensive understanding of the cognitive deficits after the acute illness allows clinicians the capacity to monitor and manage ongoing cognitive alterations appropriately. It also assists with communicating expectations of disease trajectory to patients and their caregivers. While previous studies have provided a foundation for the developing literature, to our knowledge, no studies have yet engaged in a Wechsler index-driven analysis of outcomes using the normative data provided by the test publishers. These data aim to form the basis of a cognitive knowledge repository to assist clinicians in AE patient assessment and ongoing management of cognitive impairment. Further, it may assist with understanding the possible impacted cognitive networks, which can assist with understanding disease pathogenesis and prognostication.

In this study, we aimed to characterize cognitive outcomes in AE patients, specifically assessing verbal comprehension, perceptual reasoning, working memory, processing speed, auditory memory, visual memory, immediate memory, and delayed memory indices compared to normative data. We explored differences between the total cohort, seropositive, and seronegative cohorts. Additionally, we examined patterns of intact and non-intact cognitive impairment in AE patients.

### Index characterization

This study demonstrated that in this AE cohort, tested in the chronic phase with an average time from symptom to the cognitive assessment of 39 months (3.25 years), perceptual reasoning, processing speed, and working memory abilities were significantly below normative data. The support for this was strengthened by the results of paired *t*-tests, which revealed that the VCI demonstrated a higher score than the WMI, the PRI, and the PSI. In contrast, no statistically significant differences were observed among the WMI, PRI, and PSI. When the seropositive and seronegative cohorts were analyzed separately, the processing speed index was significantly below normative data in the seropositive patients. In contrast, the working memory and processing speed indexes were significantly below the normative mean in the seronegative group.

#### Information processing speed in AE patients

In chronic stages of the disease (e.g., post 6 months), processing speed deficits are not often reported as a fundamental impairment in AE at a group level. Specifically, Guasp et al. focused on anti-NMDAR ab-mediated AE patients. They identified deficits compared to controls on the Processing Speed Index (PSI) and the TMT-A at the 1-year mark [17]. Notably, the impairment rate in this domain in anti-NMDAR ab-mediated AE patients decreased to less than 10% at the 1-year time point. Longitudinal analysis revealed significant improvements in processing speed from initial intake to 6 months, but these improvements did not persist from 6 to 12 months. Of note, the test of automatic motor speed (TMT A) did not exhibit a similar pattern, as no significant differences were observed between different time points.

Across studies, a diverse range of processing speed tests have been employed, including automatic motor processing tests such as the TMT A [5, 18], DKEFS Number Sequencing [5] and/or motor speed [7]. Significant variability in findings has emerged across subtypes, with some studies reporting no significant speed deficits compared to healthy controls in anti-NMDAR ab-mediated AE [7]. In contrast, others reported that 13% of patients presented with deficits in anti-LGI1 ab-mediated AE [18]. Additionally, at an individual level, significant variability in outcomes was observed in patients with anti-LGI1 ab-mediated AE [5]. However, none of the patients were noted as impaired across all processing speed tests employed. These diverse results underscore the complexity and heterogeneity of processing speed outcomes in AE.

The results regarding the processing speed index in AE’s chronic stages are noteworthy. This results may be attributed to a methodological factor. The current study employed WAIS-IV Coding and Symbol Search subtests [13], which can require additional cognitive load compared to a routine processing speed test, such as the TMT-A. As such, the reduced processing speed demonstrated in this study may reflect this additional cognitive load rather than a reflection of a clinical bradyphrenia presentation per se. This hypothesis gains further support from the inclusion of data from an automated motor processing speed test, the TMT-A, which was also obtained from the same cohort and is available in the appendices. The results of this test indicate that, at a group level, the total cohort was not significantly below normative data when we defined significantly below 1.5 SD below the normative mean (Table S8).

Of note was that the longer time between assessment and admission and assessment and symptom onset was significantly correlated with worse performance on processing speed tests. This is likely a reflection of selection bias. The cohort was obtained through outpatient neurology clinics. It could be suggested that patients who continue to be seen regularly in outpatient clinics many years after their initial illness have poorer outcomes than patients who were discharged. Patients who have continued to follow up may have global cognitive impairments, relapses, or perhaps significant seizure burden, which would affect processing speed.

#### Working memory in AE patients

Previous research has taken a varied approach to assessing working memory. Some studies combined executive function and working memory tests [7] or integrated basic attention and working memory tests into aggregated scores [19–21].

While our study revealed a reduced WMI, the WMI has limitations as a measure. The working memory system is a multi-component system that plays a crucial role in our ability to perform complex tasks [22]. Among its components, the central executive is particularly important for managing complex cognitive processes, such as mental arithmetic and problem-solving, which are highly representative in the subtests of the WMI—including digit span backwards, sequencing and the arithmetic subtests. Consequently, basic attention is poorly represented.

Tasks that rely heavily on working memory, such as digit span backwards and arithmetic, are closely associated with higher-order cognitive functions. Therefore, the observed decrease in working memory may not be a failure of fundamental cognitive functions, such as basic attention, but rather an indication of reduced higher-order cognitive functions, such as working memory. Either way, the former suggests further investigations into this population’s prevalence, and rates of basic attention failures are warranted. While the latter suggests, it is necessary to conduct further investigations into higher-order functions in AE as they are not well-represented in the current batteries (WAIS, WASI or WMS).

#### Verbal comprehension and perceptual reasoning in AE patients

The Verbal Comprehension Index was not significantly different from the normative data in any cohorts. In contrast, PRI was significantly below the normative data for the total cohort and the seronegative group. While the VCI and PRI are considered indices of ‘general (cognitive) ability’ in the Wechsler framework, the Cattell–Horn–Carroll theory emphasizes important distinctions between them. Namely that the VCI is heavily loaded onto crystallized knowledge, while in contrast, PRI integrates aspects of visual processing, fluid reasoning, abstract reasoning and problem-solving. This suggests that these functions are more affected in AE compared to tasks that require crystallized knowledge.

However, when interpreting these findings, it is important to consider the findings of the PSI. Slowed processing speed, whether due to fundamental processing speed impairment or slowed secondary to a build-up of executive function leading to a reduction in efficiency of information processing, could potentially influence the subtests of the PRI. One of the PRI subtests being timed makes it crucial to account for the impact of processing speed on overall PRI performance. Therefore, while a decrease in PRI scores may imply disruption in frontal network functions, the role of processing speed needs careful consideration in understandings these outcomes.

#### Wechsler Memory Scale (WMS) in AE patients

When examining the indices of the WMS (auditory memory, visual memory, immediate memory and delayed memory), surprisingly, all of the memory indices were within the normative mean for the total and seropositive cohorts. The seronegative AE cohort demonstrated delayed memory abilities below the normative mean.

The memory findings of the current study were unexpected. While a comprehensive memory assessment demonstrated that the seronegative cohort had reduced delayed memory, previous studies examining seropositive AE patients have concluded that memory is the primary cognitive impairment in these patients [2–4, 6]. The present study’s results may reflect the small cohort for each seropositive group compared to larger cohort studies, such as Heine and colleagues [4]. Heterogeneity in memory assessment tools employed across studies may also account for this discrepancy. A logico-semantic memory test (e.g. a prose test) along with a supraspan memory (e.g. a list-learning test) with semantic categories was employed in the current study. These paradigms integrate more lateral aspects of the temporal structures due to their semantic loadings. Previous studies have often used supraspan verbal learning tests, such as the RAVLT, which do not have embedded semantic categories [4, 6, 9, 23]. Without these categories, tests like the RAVLT (compared to the CVLT) rely less on semantic networks, engaging more of the mesial aspect of the hippocampus structures [24]. The inherent semantic structure present in logico-semantic tests and the supraspan test with embedded semantic structures may provide an inherent structure for recall. This may overcome, if present, the effects of an underlying dysexecutive syndrome. Previous studies have suggested that logico-semantic tests are often not associated with the severity of executive dysfunction [25]. Consequently, the current study may demonstrate that many of the memory impairments commented on in previous studies may be secondary to executive dysfunction rather than a primary amnestic syndrome. Further exploration of this hypothesis is recommended in the literature.

The finding that seropositive patients did not perform significantly below the normative mean on the memory indices is particularly intriguing, given that this subgroup primarily consists of individuals with anti-NMDAR and anti-LGI1 antibodies, two autoimmune encephalitis groups often associated with memory deficits [5, 7, 8, 26–29]. One potential explanation for these results could be related to the sample recruitment process, where participants were not specifically selected based on reported cognitive difficulties but were enrolled due to having an AE diagnosis. This may indicate that this cohort reflects real-life impairment rates, suggesting a less pronounced cognitive impact than previously indicated in the literature.

Although the seronegative subgroup demonstrated significantly poorer performances than normative data, no significant differences were observed between the seropositive and seronegative groups. The smaller sample size, halved due to subgroup analysis, may have limited our sensitivity to detect subtle changes between these groups. Future studies should address this limitation by employing larger sample sizes to effectively identify potential differences between the seropositive and seronegative AE patients.

It is important to note that the lack of significant group differences does not negate the possibility of individual variability in cognitive outcomes. For instance, within the anti-LGI1 ab-mediated group, patients can exhibit hippocampal changes associated with poorer memory outcomes in this subgroup [30]. Consequently, these individual factors might offset each other at a group level, resulting in a lack of significant differences between the seropositive and seronegative groups. However, some patients may still experience significant cognitive impairments at an individual level. The consideration of differences between the individual and at a group level underscores the complexity of cognitive outcomes in AE. It highlights the need for further exploration of factors contributing to cognitive outcomes.

Finally, while there are several strengths to using the WMS-IV, its use as a measure of memory function also has limitations, primarily stemming from the design of its indices. One notable limitation is the scale’s limited sensitivity in detecting subtle changes in memory function due to its integration of subtests into indices. Furthermore, the WMS-IV’s design may not comprehensively capture or precisely assess specific memory functions, such as supraspan memory, logico-semantic memory and visual memory. By integrating these functions into indices, their unique characteristics and contributions may be diluted or overshadowed, leading to potential limitations in accurately evaluating these specific memory domains. The heterogeneity within the indices also poses another challenge. Since indices are composite measures derived from multiple tests or subtests, significant variability in performance across the included tests can arise. Finally, the WMS does not evaluate retrograde amnesia. Previous studies have indicated that limbic encephalitis has been associated with retrograde memory deficits, often affecting autobiographical memory [2]. Given these limitations, further exploration of memory function is warranted, employing tests that specifically target different memory functions, including autobiographical–episodic memory.

Unexpectedly, there were significant differences between the performances of males and females on the IMI, DMI and VMI, with men outperforming females. While the male cohort was likelier to be older, the finding is unusual, as the normative data are stratified by age. Of note, this reflects similar results from the consortium’s previous retrospective study, which indicate that while not significant, being a male was a predictor of intact cognition after AE, with one of the larger effect sizes of all the variables explored [31]. This finding has yet to arise in other studies, with previous systematic reviews concluding that sex is unlikely to have significant prognostic value [32]. Further exploration of this outcome is recommended in future studies.

### Patterns of cognitive impairment

Pattern analysis allows for the identification of potential cognitive profiles. It can provide insight into the interrelationships between different domains, thus offering a comprehensive understanding of cognitive functioning compared to traditional single-test analysis.

When we explored patterns of impairment, of 256 possible patterns, 11 patterns were observed in the data set. The most frequent pattern observed in the data was preserved cognition. The three subsequent most common patterns observed were: (1) isolated visual memory impairment, (2) isolated processing speed impairment, (3) auditory memory and delayed memory impairment. Of note was the isolated visual memory impairment observed. This unusual finding of an isolated domain, heavily loaded to visuospatial/ visuoconstructional abilities, mirrors a similar result in a retrospective analysis that retrospectively examined patients with a diagnosis of possible AE [31]. However, the visual reproduction test can potentially be a sensitive measure of memory, as verbal memory strategies, such as labelling components, are commonly employed during visual memory tasks [33]. These strategies enhance memory recall without relying on semantic structures or associations. As a result, performance on the visual reproduction test may indicate difficulties in memory functioning. Further prospective studies should examine these findings in more detail by, for example, employing qualitative descriptions of Rey Complex Figure Test copies [34].

The preliminary findings of this analysis suggest positive long-term cognitive outcomes in patients with AE. Secondarily, they indicate a significant heterogeneity and a multitude of patterns in cognitive outcomes, suggesting a wide variation among individuals. These results imply that cognitive outcomes after AE may be non-specific. Consequently, this could suggest that there are widely distributed pathological processes underlying cognitive impairments. The complexity observed in the cognitive profiles highlights the need for further investigation to understand better the underlying mechanisms contributing to the diverse cognitive outcomes in AE.

### Limitations

Several limitations are to be noted. One of the primary limitations pertains to using the WASI, WAIS and WMS batteries. While these batteries offer well-established reliability, a comprehensive and specific assessment of cognitive domains, robust criterion-related validity, and narrow confidence intervals, they also have inherent limitations. Many of these limitations are noted in the WMS section of this discussion. As highlighted, these scales were primarily designed to assess intellectual functioning, limiting their sensitivity in detecting subtle changes in cognitive functions due to their integration of subtests into indices. Additionally, integrating various cognitive functions into these indices may dilute or overshadow the unique contributions of each function. While this analysis serves as an initial step, it is imperative to conduct further investigations using targeted assessments of specific functions, such as executive functions, or employing different paradigms of memory assessment to gain a more comprehensive understanding of the cognitive profiles in AE.

Patients who were severely cognitively or physically impaired could not be included in these analyses as they could not complete aspects of the WAIS or WMS scales. Further, due to the COVID-19 pandemic restrictions at the time of recruitment, the current cohort reflects patients who were not residing in nursing home/care facilities and hence could travel to the hospital to perform the cognitive assessment. Consequently, the current profiles only reflect patients who engaged fully in extensive neuropsychological assessment and do not reflect those who are highly impaired and rely on carers for day-to-day functioning. In addition, the memory tests explored in the current study do not explore what benefits, if any, occur with a recognition format. An in-depth exploration of memory profiles is warranted to elucidate the cognitive outcomes of this disease. This study did not explore variables that can have deleterious effects on cognition—seizures, anti-seizure medication, psychopathology and fatigue [35]. An in-depth characterization of these factors and their correlation to cognitive outcomes would of interest for future studies. While the sample size does represent a large proportion of patients with this disease in Victoria, Australia, it is limited. This is particularly evident in the AE subtype analyses, which may be underpowered.

Further analysis with a larger cohort for each subtype is warranted. Finally, we did not correct for multiple comparisons for the AE sub-type analysis due to small sample sizes, which increases the risk of false-positive findings and the possibility of obtaining statistically significant results by chance. This can affect the validity and generalizability of the findings and should be considered when interpreting the results.

## Conclusions

The present study reveals significant deficits in perceptual reasoning, processing speed, and working memory among patients in the chronic phase of AE. Specifically, seropositive AE patients exhibited below-norm processing speed, while both seropositive and seronegative patients demonstrated significantly reduced working memory and processing speed. Delayed memory was significantly below expectations only in seronegative patients.

Notably, pattern analysis revealed that long-term cognitive outcomes are positive for many patients. In those patients who have ongoing cognitive difficulties, these outcomes are heterogeneous. This complexity suggests that cognitive outcomes may vary widely among individuals.

It is important to note that patients with severe cognitive impairment were excluded from these analyses. While this excludes a number of patients after AE, the findings here pertain to a cohort of AE patients typically encountered in clinical neuropsychology settings. As such, our results can be extrapolated to other AE patients in clinic, highlighting the importance of thoroughly assessing these cognitive functions during neuropsychology consultations.

Based on the findings of the perceptual reasoning index and working memory index, further prospective investigation of tests assessing executive function in the AE population is warranted. Such exploration may deepen our understanding of executive function deficits in AE and potentially inform targeted interventions to address these specific cognitive challenges.

### Supplementary Information

Below is the link to the electronic supplementary material.

Supplementary file1 (DOCX 142 KB)

## Data Availability

Data was not provided in the article because of space limitations may be shared (anonymized) at the request of any qualified investigator for purposes of replicating procedures and results.

## References

[1] Graus F, Titulaer MJ, Balu R, Benseler S, Bien CG, Cellucci T (2016). A clinical approach to diagnosis of autoimmune encephalitis. Lancet Neurol.

[2] Witt JA, Helmstaedter C (2021). Neuropsychological evaluations in limbic encephalitis. Brain Sci.

[3] Griffith SP, Malpas CB, Kyndt C, Alpitsis R, O’Brien TJ, Monif M (2021). Cognition and psychopathology in autoimmune encephalitides: a focus on risk factors and patient outcomes. Clin Exp Neuroimmunol.

[4] Heine J, Kopp UA, Klag J, Ploner CJ, Prüss H, Finke C (2021). Long-term cognitive outcome in anti-*N*-methyl-d-aspartate receptor encephalitis. Ann Neurol.

[5] Galioto R, Aboseif A, Krishnan K, Lace J, Kunchok A (2022). Cognitive outcomes in anti-LGI-1 encephalitis. J Int Neuropsychol Soc.

[6] Mueller C, Langenbruch LM, Rau JMH, Brix T, Strippel C, Dik A (2021). Determinants of cognition in autoimmune limbic encephalitis—a retrospective cohort study. Hippocampus.

[7] McKeon GL, Scott JG, Spooner DM, Ryan AE, Blum S, Gillis D (2016). Cognitive and social functioning deficits after anti-*N*-methyl-d-aspartate receptor encephalitis: an exploratory case series. J Int Neuropsychol Soc.

[8] Finke C, Kopp UA, Prüss H, Dalmau J, Wandinger KPP, Ploner CJ (2012). Cognitive deficits following anti-NMDA receptor encephalitis. J Neurol Neurosurg Psychiatry.

[9] McKeon GL, Robinson GA, Ryan AE, Blum S, Gillis D, Finke C (2018). Cognitive outcomes following anti- * N * -methyl-  d  -aspartate receptor encephalitis: a systematic review. J Clin Exp Neuropsychol.

[10] Lichtenberger EO, Kaufman AS (2012). Essentials of WAIS-IV assessment.

[11] Wechsler D (2009). Wechsler Memory Scale—fourth edition (WMS–IV) technical and interpretive manual.

[12] Wechsler D (2011). Wechsler Abbreviated Scale of Intelligence—second edition (WASI-II).

[13] Wechsler D (2008). Wechsler Adult Intelligence Scale—fourth edition (WAIS-IV).

[14] Woods SP, Delis DC, Scott JC, Kramer JH, Holdnack JA (2006). The California Verbal Learning Test-second edition: test–retest reliability, practice effects, and reliable change indices for the standard and alternate forms. Arch Clin Neuropsychol.

[15] Drozdick LW, Holdnack JA, Hilsabeck RC (2011). Essentials of WMS-IV assessment.

[16] Binks SNM, Veldsman M, Easton A, Leite MI, Okai D, Husain M, Irani SR (2021). Residual fatigue and cognitive deficits in patients after leucine-rich glioma-inactivated 1 antibody encephalitis. JAMA Neurol.

[17] Guasp M, Rosa-Justicia M, Muñoz-Lopetegi A, Martínez-Hernández E, Armangué T, Sugranyes G (2022). Clinical characterisation of patients in the post-acute stage of anti-NMDA receptor encephalitis: a prospective cohort study and comparison with patients with schizophrenia spectrum disorders. Lancet Neurol.

[18] Rodriguez A, Klein CJ, Sechi E, Alden E, Basso MR, Pudumjee S (2022). LGI1 antibody encephalitis: acute treatment comparisons and outcome. J Neurol Neurosurg Psychiatry.

[19] Frisch C, Malter MP, Elger CE, Helmstaedter C (2013). Neuropsychological course of voltage-gated potassium channel and glutamic acid decarboxylase antibody related limbic encephalitis. Eur J Neurol.

[20] Bettcher BM, Gelfand JM, Irani SR, Neurol M, Geschwind MD (2015). More than memory impairment in voltage-gated potassium channel complex encephalopathy. Eur J Neurol.

[21] van Sonderen A, Coenders EC, Sanchez E, De MAAM, Van MH, DeWirtz FW (2016). Anti-LGI1 encephalitis: clinical syndrome and long-term follow-up. Neurology.

[22] Baddeley A (2007). Working memory, thought, and action.

[23] Finke C, Pruss H, Heine J, Reuter S, Kopp UA, Wegner F (2017). Evaluation of cognitive deficits and structural hippocampal damage in encephalitis with leucine-rich, glioma-inactivated 1 antibodies. JAMA Neurol.

[24] Saling MM (2009). Verbal memory in mesial temporal lobe epilepsy: beyond material specificity. Brain J Neurol.

[25] Tremont G, Halpert S, Javorsky DJ, Stern RA (2000). Differential impact of executive dysfunction on verbal list learning and story recall. Clin Neuropsychol.

[26] Heine J, Kopp UA, Klag J, Ploner CJ, Prüss H, Finke C (2021). Long-term cognitive outcome in anti-NMDA receptor encephalitis. Ann Neurol.

[27] Malter MP, Frisch C, Schoene-Bake JC, Helmstaedter C, Wandinger KP, Stoecker W (2014). Outcome of limbic encephalitis with VGKC-complex antibodies: relation to antigenic specificity. J Neurol.

[28] Szots M, Marton A, Kover F, Kiss T, Berki T, Nagy F (2014). Natural course of LGI1 encephalitis: 3–5 years of follow-up without immunotherapy. J Neurol Sci.

[29] Li Z, Cui T, Shi W, Wang Q (2016) Clinical analysis of leucine-rich glioma inactivated-1 protein antibody associated with limbic encephalitis onset with seizures. Medicine (United States) 95(28) 10.1097/MD.0000000000004244 PMC4956827 27428233

[30] Miller TD, Chong TTJ, Aimola Davies AM, Ng TWC, Johnson MR, Irani SR (2017). Focal CA3 hippocampal subfield atrophy following LGI1 VGKC-complex antibody limbic encephalitis. Brain.

[31] Griffith S, Wesselingh R, Broadley J, O’Shea M, Kyndt C, Meade C (2022). Psychometric deficits in autoimmune encephalitis: a retrospective study from the Australian Autoimmune Encephalitis Consortium. Eur J Neurol.

[32] Broadley J, Seneviratne U, Beech P, Buzzard K, Butzkueven H, O’Brien T (2019). Prognosticating autoimmune encephalitis: a systematic review. J Autoimmun.

[33] Lambrecq V, Alonso I, Hasboun D, Dinkelacker V, Davachi L, Samson S et al (2023) Memory functioning after hippocampal removal: does side matter? J Neuropsychol 10.1111/jnp.12309 36861271

[34] Meyers JE, Meyers KR (1995) Rey complex figure test and recognition trial. Psychological Assessment Resources, Incorporated

[35] Witt JA, Helmstaedter C (2017). How can we overcome neuropsychological adverse effects of antiepileptic drugs?. Expert Opin Pharmacother.

