# Supplementary material for: Characterizing cognitive function in patients with autoimmune encephalitis: an Australian prospective study
Source: J Neurol. 2023 Sep 14;271(1):310–24. doi: 10.1007/s00415-023-11967-w (PMC10770222; doi:10.1007/s00415-023-11967-w)
Supplement: Supplementary file 1 — Supplementary file1 (DOCX 142 KB) [file 415_2023_11967_MOESM1_ESM.docx]

**Supplementary Data**

|  |
| --- |

**Table S1. Subtests Descriptive Data**

|  | WASI - BD | WASI- MR | WASI - SIM | WASI - VOCAB | WMS - LM1 | WMS - LM2 | WMS - VR1 | WMS - VR2 | CVLT Total | CVLT Long Free Recall |
| --- | --- | --- | --- | --- | --- | --- | --- | --- | --- | --- |
| N | 40 | 45 | 44 | 45 | 46 | 46 | 45 | 45 | 46 | 46 |
|  |  |  |  |  |  |  |  |  |  |  |
| Minimum | -2.0 | -2.9 | -2.3 | -2.1 | -2.7 | -3.1 | -3.1 | -3.1 | -2.1 | -4.5 |
| Maximum | 2.1 | 1.3 | 2.3 | 3.0 | 1.0 | 1.3 | 2.0 | 2.7 | 2.2 | 2.0 |
| Range | 4.1 | 4.2 | 4.6 | 5.1 | 3.7 | 4.4 | 5.1 | 5.8 | 4.3 | 6.5 |
|  |  |  |  |  |  |  |  |  |  |  |
| Mean | -0.15 | -0.35 | 0.24 | 0.17 | -0.24 | -0.54 | -0.12 | -0.22 | 0.083 | -0.35 |
| Std. Deviation | 0.92 | 0.92 | 0.98 | 1.0 | 0.96 | 1.2 | 1.3 | 1.6 | 1.2 | 1.5 |
| Std. Error of Mean | 0.15 | 0.14 | 0.15 | 0.15 | 0.14 | 0.18 | 0.19 | 0.23 | 0.17 | 0.22 |
|  |  |  |  |  |  |  |  |  |  |  |
| Lower 95% CI of mean | -0.44 | -0.63 | -0.060 | -0.13 | -0.52 | -0.90 | -0.50 | -0.70 | -0.26 | -0.80 |
| Upper 95% CI of mean | 0.15 | -0.075 | 0.54 | 0.48 | 0.046 | -0.18 | 0.26 | 0.25 | 0.43 | 0.10 |

|  | WAIS DS | WAIS AR | WAIS SS | WAIS CD |
| --- | --- | --- | --- | --- |
| Number of values | 47 | 45 | 40 | 41 |
|  |  |  |  |  |
| Minimum | -2.0 | -2.0 | -3.1 | -2.7 |
| Maximum | 10 | 2.3 | 3.0 | 1.0 |
| Range | 12 | 4.3 | 6.1 | 3.7 |
|  |  |  |  |  |
| Mean | -0.12 | -0.21 | -0.25 | -0.60 |
| Std. Deviation | 1.8 | 1.0 | 1.2 | 0.83 |
| Std. Error of Mean | 0.26 | 0.15 | 0.19 | 0.13 |
|  |  |  |  |  |
| Lower 95% CI of mean | -0.64 | -0.51 | -0.63 | -0.86 |
| Upper 95% CI of mean | 0.41 | 0.098 | 0.12 | -0.34 |

**Table S2. Paired sample t -tests**

| **Comparison** | **Measure 1** | | **Measure 2** | | **t(df)** | **p** |
| --- | --- | --- | --- | --- | --- | --- |
|  | **M** | **SD** | **M** | **SD** |  |  |
| VCI & PRI | 103.00 | 14.37 | 94.72 | 14.53 | 2.40 | 0.02 |
| VCI & VMI | 103.00 | 14.37 | 94.26 | 14.97 | 3.40 | 0.001 |
| VCI & PSI | 103.00 | 14.37 | 93.43 | 13.91 | 2.82 | 0.008 |
| PRI & WMI | 94.72 | 14.53 | 94.26 | 14.97 | 0.72 | 0.48 |
| PRI & PSI | 94.72 | 14.53 | 93.43 | 13.91 | 0.27 | 0.79 |
| PSI & WMI | 93.43 | 13.91 | 94.26 | 14.97 | 0.26 | 0.78 |
| AMI & VMI | 95.74 | 16.58 | 98.95 | 21.18 | -0.98 | 0.33 |
| AMI & IMI | 95.74 | 16.58 | 97.28 | 14.70 | -0.93 | 0.359 |
| AMI & DMI | 95.74 | 16.58 | 95.54 | 20.50 | 0.13 | 0.895 |
| VMI & IMI | 98.95 | 21.18 | 97.28 | 14.70 | 0.80 | 0.43 |
| VMI & DMI | 98.95 | 21.18 | 95.54 | 20.50 | 1.34 | 0.187 |
| IMI & DMI | 97.28 | 14.70 | 95.54 | 20.50 | 0.85 | 0.398 |

**Table S3. Seronegative vs. Seropositive**

| Independent Samples T-Test | | | | | | | | | | | | | |
| --- | --- | --- | --- | --- | --- | --- | --- | --- | --- | --- | --- | --- | --- |
|  | | | | | | | | | | **95% CI for Cohen's d** | | | |
|  | | **Statistic** | | **df** | | **p** | | **Cohen's d** | | **Lower** | | **Upper** | |
| VCI |  | -0.416 |  | 42 |  | 0.679 |  | -0.126 |  | -0.717 |  | 0.467 |  |
| PRI |  | -0.532 |  | 37 |  | 0.598 |  | -0.170 |  | -0.798 |  | 0.460 |  |
| WMI | * | 233.50 |  |  |  | 0.506 |  | -0.116 |  | -0.425 |  | 0.218 |  |
| PSI |  | 0.281 |  | 38 |  | 0.780 |  | 0.089 |  | -0.532 |  | 0.708 |  |
| AMI |  | -1.541 |  | 41 |  | 0.131 |  | -0.470 |  | -1.074 |  | 0.139 |  |
| VMI |  | -0.964 |  | 41 |  | 0.341 |  | -0.294 |  | -0.894 |  | 0.309 |  |
| IMI |  | -1.257 |  | 41 |  | 0.216 |  | -0.383 |  | -0.985 |  | 0.223 |  |
| DMI |  | -1.931 |  | 41 |  | 0.060 |  | -0.589 |  | -1.197 |  | 0.026 |  |
|  | | | | | | | | | | | | | |

*Note. Student T test reported unless otherwise indicated. *Shapiro-Wilks significant (p<.05). Mann-Whitney U Test reported. Abbreviations: VCI = Verbal Comprehension Index; PRI = Perceptual Reasoning Index; WMI = Working Memory Index; PSI = Processing Speed Index; AMI = Auditory Memory Index; VMI = Visual Memory Index; IMI= Immediate Memory Index; DMI = Delayed Memory Index*

|  |  |  |  |  |  |  |  |  |  |  |  |
| --- | --- | --- | --- | --- | --- | --- | --- | --- | --- | --- | --- |
| Seronegative and Seropositive Descriptive Statistics | | | | | | | | | | | |
|  | | **Group** | | **N** | | **Mean** | | **SD** | | **SE** | |
| VCI |  | Seronegative |  | 23 |  | 102.130 |  | 16.120 |  | 3.361 |  |
|  |  | Seropositive |  | 21 |  | 103.952 |  | 12.492 |  | 2.726 |  |
| PRI |  | Seronegative |  | 20 |  | 93.500 |  | 15.545 |  | 3.476 |  |
|  |  | Seropositive |  | 19 |  | 96.000 |  | 13.675 |  | 3.137 |  |
| WMI |  | Seronegative |  | 24 |  | 93.000 |  | 16.566 |  | 3.382 |  |
|  |  | Seropositive |  | 22 |  | 95.636 |  | 13.265 |  | 2.828 |  |
| PSI |  | Seronegative |  | 20 |  | 94.050 |  | 15.240 |  | 3.408 |  |
|  |  | Seropositive |  | 20 |  | 92.800 |  | 12.800 |  | 2.862 |  |
| AMI |  | Seronegative |  | 22 |  | 92.000 |  | 18.252 |  | 3.891 |  |
|  |  | Seropositive |  | 21 |  | 99.667 |  | 13.987 |  | 3.052 |  |
| VMI |  | Seronegative |  | 22 |  | 95.909 |  | 21.893 |  | 4.668 |  |
|  |  | Seropositive |  | 21 |  | 102.143 |  | 20.431 |  | 4.458 |  |
| IMI |  | Seronegative |  | 22 |  | 94.545 |  | 14.783 |  | 3.152 |  |
|  |  | Seropositive |  | 21 |  | 100.143 |  | 14.402 |  | 3.143 |  |
| DMI |  | Seronegative |  | 22 |  | 89.818 |  | 21.930 |  | 4.675 |  |
|  |  | Seropositive |  | 21 |  | 101.524 |  | 17.443 |  | 3.806 |  |

*Note. Abbreviations: VCI = Verbal Comprehension Index; PRI = Perceptual Reasoning Index; WMI = Working Memory Index; PSI = Processing Speed Index; AMI = Auditory Memory Index; VMI = Visual Memory Index; IMI= Immediate Memory Index; DMI = Delayed Memory Index*

**Table S4. NMDAR vs. LGI1**

| Independent Samples T-Test | | | | | | | | | | | | | |
| --- | --- | --- | --- | --- | --- | --- | --- | --- | --- | --- | --- | --- | --- |
|  | | | | | | | | | | **95% CI for Cohen's d** | | | |
|  | | **Statistic** | | **df** | | **p** | | **Cohen's d** | | **Lower** | | **Upper** | |
| VCI |  | -1.014 |  | 14 |  | 0.328 |  | -0.511 |  | -1.508 |  | 0.503 |  |
| PRI |  | 0.241 |  | 12 |  | 0.813 |  | 0.135 |  | -0.963 |  | 1.226 |  |
| WMI |  | 30.00 |  |  |  | 0.593 |  | -0.167 |  | -0.626 |  | 0.378 |  |
| PSI |  | -0.696 |  | 13 |  | 0.499 |  | -0.381 |  | -1.457 |  | 0.709 |  |
| AMI |  | 1.348 |  | 14 |  | 0.199 |  | 0.679 |  | -0.351 |  | 1.687 |  |
| VMI |  | -0.057 |  | 14 |  | 0.955 |  | -0.029 |  | -1.016 |  | 0.960 |  |
| IMI |  | 0.303 |  | 14 |  | 0.766 |  | 0.153 |  | -0.839 |  | 1.139 |  |
| DMI |  | 1.210 |  | 14 |  | 0.246 |  | 0.610 |  | -0.413 |  | 1.612 |  |
|  | | | | | | | | | | | | | |
| Note.  Student's t-test unless otherwise noted. *Levene test is significant (p < .05), Mann-Whitney U test reported. *Abbreviations: VCI = Verbal Comprehension Index; PRI = Perceptual Reasoning Index; WMI = Working Memory Index; PSI = Processing Speed Index; AMI = Auditory Memory Index; VMI = Visual Memory Index; IMI= Immediate Memory Index; DMI = Delayed Memory Index.* | | | | | | | | | | | | | |

| Group Descriptive | | | | | | | | | | | |
| --- | --- | --- | --- | --- | --- | --- | --- | --- | --- | --- | --- |
|  | | **Group** | | **N** | | **Mean** | | **SD** | | **SE** | |
| VCI |  | NMDAR |  | 9 |  | 102.222 |  | 11.054 |  | 3.685 |  |
|  |  | LGI1 |  | 7 |  | 109.143 |  | 16.283 |  | 6.154 |  |
| PRI |  | NMDAR |  | 9 |  | 97.667 |  | 14.620 |  | 4.873 |  |
|  |  | LGI1 |  | 5 |  | 95.600 |  | 16.727 |  | 7.481 |  |
| WMI |  | NMDAR |  | 9 |  | 91.778 |  | 7.412 |  | 2.471 |  |
|  |  | LGI1 |  | 8 |  | 100.000 |  | 20.354 |  | 7.196 |  |
| PSI |  | NMDAR |  | 10 |  | 91.500 |  | 13.168 |  | 4.164 |  |
|  |  | LGI1 |  | 5 |  | 96.800 |  | 15.418 |  | 6.895 |  |
| AMI |  | NMDAR |  | 9 |  | 105.111 |  | 12.313 |  | 4.104 |  |
|  |  | LGI1 |  | 7 |  | 94.714 |  | 18.554 |  | 7.013 |  |
| VMI |  | NMDAR |  | 9 |  | 101.333 |  | 24.890 |  | 8.297 |  |
|  |  | LGI1 |  | 7 |  | 102.000 |  | 20.881 |  | 7.892 |  |
| IMI |  | NMDAR |  | 9 |  | 102.444 |  | 15.749 |  | 5.250 |  |
|  |  | LGI1 |  | 7 |  | 100.000 |  | 16.371 |  | 6.188 |  |
| DMI |  | NMDAR |  | 9 |  | 106.556 |  | 19.236 |  | 6.412 |  |
|  |  | LGI1 |  | 7 |  | 94.714 |  | 19.653 |  | 7.428 |  |
|  | | | | | | | | | | | |

*Abbreviations: VCI = Verbal Comprehension Index; PRI = Perceptual Reasoning Index; WMI = Working Memory Index; PSI = Processing Speed Index; AMI = Auditory Memory Index; VMI = Visual Memory Index; IMI= Immediate Memory Index; DMI = Delayed Memory Index*

**Table S5. NMDAR vs. Rest of Seropositive Cohort**

| Independent Samples T-Test | | | | | | | | | | | | | |
| --- | --- | --- | --- | --- | --- | --- | --- | --- | --- | --- | --- | --- | --- |
|  | | | | | | | | | | **95% CI for Cohen's d** | | | |
|  | | **Statistic** | | **df** | | **p** | | **Cohen's d** | | **Lower** | | **Upper** | |
| VCI |  | -0.540 |  | 19 |  | 0.596 |  | -0.238 |  | -1.102 |  | 0.633 |  |
| PRI* |  | 52.00 |  |  |  | 0.595 |  | 0.156 |  | -0.360 |  | 0.598 |  |
| WMI* |  | 47.50 |  |  |  | 0.479 |  | -0.188 |  | -0.599 |  | 0.302 |  |
| PSI |  | -0.444 |  | 18 |  | 0.662 |  | -0.199 |  | -1.075 |  | 0.683 |  |
| AMI |  | 1.604 |  | 19 |  | 0.125 |  | 0.707 |  | -0.194 |  | 1.591 |  |
| VMI |  | -0.153 |  | 19 |  | 0.880 |  | -0.068 |  | -0.931 |  | 0.798 |  |
| IMI |  | 0.624 |  | 19 |  | 0.540 |  | 0.275 |  | -0.597 |  | 1.140 |  |
| DMI |  | 1.154 |  | 19 |  | 0.263 |  | 0.509 |  | -0.377 |  | 1.382 |  |
|  | | | | | | | | | | | | | |
| *Note. Student T test reported unless otherwise indicated. *Shapiro-Wilks significant (p<.05). Mann-Whitney U Test reported. Abbreviations: VCI = Verbal Comprehension Index; PRI = Perceptual Reasoning Index; WMI = Working Memory Index; PSI = Processing Speed Index; AMI = Auditory Memory Index; VMI = Visual Memory Index; IMI= Immediate Memory Index; DMI = Delayed Memory Index* | | | | | | | | | | | | | |

| Group Descriptive | | | | | | | | | | | |
| --- | --- | --- | --- | --- | --- | --- | --- | --- | --- | --- | --- |
|  | | **Group** | | **N** | | **Mean** | | **SD** | | **SE** | |
| VCI |  | NMDAR |  | 9 |  | 102.222 |  | 11.054 |  | 3.685 |  |
|  |  | Other Seropositive |  | 12 |  | 105.250 |  | 13.805 |  | 3.985 |  |
| PRI |  | NMDAR |  | 9 |  | 97.667 |  | 14.620 |  | 4.873 |  |
|  |  | Other Seropositive |  | 10 |  | 94.500 |  | 13.369 |  | 4.228 |  |
| WMI |  | NMDAR |  | 9 |  | 91.778 |  | 7.412 |  | 2.471 |  |
|  |  | Other Seropositive |  | 13 |  | 98.308 |  | 15.887 |  | 4.406 |  |
| PSI |  | NMDAR |  | 10 |  | 91.500 |  | 13.168 |  | 4.164 |  |
|  |  | Other Seropositive |  | 10 |  | 94.100 |  | 12.991 |  | 4.108 |  |
| AMI |  | NMDAR |  | 9 |  | 105.111 |  | 12.313 |  | 4.104 |  |
|  |  | Other Seropositive |  | 12 |  | 95.583 |  | 14.248 |  | 4.113 |  |
| VMI |  | NMDAR |  | 9 |  | 101.333 |  | 24.890 |  | 8.297 |  |
|  |  | Other Seropositive |  | 12 |  | 102.750 |  | 17.535 |  | 5.062 |  |
| IMI |  | NMDAR |  | 9 |  | 102.444 |  | 15.749 |  | 5.250 |  |
|  |  | Other Seropositive |  | 12 |  | 98.417 |  | 13.754 |  | 3.970 |  |
| DMI |  | NMDAR |  | 9 |  | 106.556 |  | 19.236 |  | 6.412 |  |
|  |  | Other Seropositive |  | 12 |  | 97.750 |  | 15.743 |  | 4.545 |  |

*Abbreviations: VCI = Verbal Comprehension Index; PRI = Perceptual Reasoning Index; WMI = Working Memory Index; PSI = Processing Speed Index; AMI = Auditory Memory Index; VMI = Visual Memory Index; IMI= Immediate Memory Index; DMI = Delayed Memory Index*

**Table S6. LGI-1 Antibody vs. other seropositive.**

| Independent Samples T-Test | | | | | | | | | | | | | |
| --- | --- | --- | --- | --- | --- | --- | --- | --- | --- | --- | --- | --- | --- |
|  | | | | | | | | | | **95% CI for Cohen's d** | | | |
|  | | **Statistic** | | **df** | | **p** | | **Cohen's d** | | **Lower** | | **Upper** | |
| VCI |  | 1.376 |  | 19 |  | 0.185 |  | 0.637 |  | -0.300 |  | 1.558 |  |
| PRI* |  | 33.00 |  |  |  | 0.889 |  | -0.057 |  | -0.580 |  | 0.499 |  |
| WMI* |  | 59.50 |  |  |  | 0.839 |  | 0.063 |  | -0.422 |  | 0.519 |  |
| PSI |  | 0.799 |  | 18 |  | 0.435 |  | 0.413 |  | -0.614 |  | 1.428 |  |
| AMI |  | -1.157 |  | 19 |  | 0.262 |  | -0.536 |  | -1.452 |  | 0.394 |  |
| VMI |  | -0.022 |  | 19 |  | 0.983 |  | -0.010 |  | -0.917 |  | 0.897 |  |
| IMI |  | -0.031 |  | 19 |  | 0.975 |  | -0.015 |  | -0.922 |  | 0.893 |  |
| DMI |  | -1.285 |  | 19 |  | 0.214 |  | -0.595 |  | -1.514 |  | 0.339 |  |
|  | | | | | | | | | | | | | |
| *Note. Student T test reported unless otherwise indicated. *Shapiro-Wilks and Levene’s tests significant (p<.05). Mann-Whitney U Test reported.*  *Abbreviations: VCI = Verbal Comprehension Index; PRI = Perceptual Reasoning Index; WMI = Working Memory Index; PSI = Processing Speed Index; AMI = Auditory Memory Index; VMI = Visual Memory Index; IMI= Immediate Memory Index; DMI = Delayed Memory Index* | | | | | | | | | | | | | |

|  | | **Group** | | **N** | | **Mean** | | **SD** | | **SE** | |
| --- | --- | --- | --- | --- | --- | --- | --- | --- | --- | --- | --- |
| VCI |  | LGI1 |  | 7 |  | 109.143 |  | 16.283 |  | 6.154 |  |
|  |  | Other Seropositive |  | 14 |  | 101.357 |  | 9.795 |  | 2.618 |  |
| PRI |  | LGI1 |  | 5 |  | 95.600 |  | 16.727 |  | 7.481 |  |
|  |  | Other Seropositive |  | 14 |  | 96.143 |  | 13.143 |  | 3.513 |  |
| WMI |  | LGI1 |  | 8 |  | 100.000 |  | 20.354 |  | 7.196 |  |
|  |  | Other Seropositive |  | 14 |  | 93.143 |  | 6.538 |  | 1.747 |  |
| PSI |  | LGI1 |  | 5 |  | 96.800 |  | 15.418 |  | 6.895 |  |
|  |  | Other Seropositive |  | 15 |  | 91.467 |  | 12.118 |  | 3.129 |  |
| AMI |  | LGI1 |  | 7 |  | 94.714 |  | 18.554 |  | 7.013 |  |
|  |  | Other Seropositive |  | 14 |  | 102.143 |  | 11.058 |  | 2.955 |  |
| VMI |  | LGI1 |  | 7 |  | 102.000 |  | 20.881 |  | 7.892 |  |
|  |  | Other Seropositive |  | 14 |  | 102.214 |  | 20.999 |  | 5.612 |  |
| IMI |  | LGI1 |  | 7 |  | 100.000 |  | 16.371 |  | 6.188 |  |
|  |  | Other Seropositive |  | 14 |  | 100.214 |  | 13.979 |  | 3.736 |  |
| DMI |  | LGI1 |  | 7 |  | 94.714 |  | 19.653 |  | 7.428 |  |
|  |  | Other Seropositive |  | 14 |  | 104.929 |  | 15.886 |  | 4.246 |  |
|  | | | | | | | | | | | |

*Abbreviations: VCI = Verbal Comprehension Index; PRI = Perceptual Reasoning Index; WMI = Working Memory Index; PSI = Processing Speed Index; AMI = Auditory Memory Index; VMI = Visual Memory Index; IMI= Immediate Memory Index; DMI = Delayed Memory Index*

**Table S7. Independent T Test - Clinical and Demographic variables by sex**

|  | | **t** | **df** | | **p** | |
| --- | --- | --- | --- | --- | --- | --- |
| Age |  | -2.371 | 48 | 0.022 | |  |
| Sex, male (N (%)) |  | 0.713 | 47 | 0.479 | |  |
| Months between symptom onset and neuropsychological assessment |  | 0.791 | 47 | 0.433 | |  |
| Months between hospital admission and neuropsychological assessment |  | -0.330 | 48 | 0.743 | |  |
| Symptom onset to Hospital admission |  | -0.103 | 47 | 0.918 | |  |
| Education, years |  | -0.370 | 48 | 0.713 | |  |
| Seropositive, y (N(%)) |  | 0.267 | 48 | 0.791 | |  |
| ASM Use, y (N(%)) |  | 1.185 | 46 | 0.242 | |  |
| Treatment Line, 1^st^ |  | NaN |  |  | |  |
| Treatment Line, y (N(%)) (2^nd^) |  | -0.693 | 47 | 0.492 | |  |
| Treatment Line, 3rd |  | NaN |  |  | |  |
| mRS at discharge (N(%)) |  | -0.414 | 44 | 0.681 | |  |
| ICU Admission during main hospital admission, y (N(%)) |  | 0.861 | 44 | 0.394 | |  |

**Table S8.**

|  | | **TMT A** |
| --- | --- | --- |
| N |  | 43 |
| Mean |  | -0.101 |
| Std. Deviation |  | 1.665 |
| Minimum |  | -7.340 |
| Maximum |  | 1.780 |
| % Impaired at 1.5 |  | 13.95 |

*Note.* TMT = Trail Making Test

**Relationship between time and cognitive outcome**

We conducted linear regression analysis to investigate the relationship between time since symptom onset and recruitment, and cognitive outcome. These analyses were non-significant. Figure S1 presents a scatterplot and reporting of the regression analysis.

**Figure S1.**

**Appendix: Australian Autoimmune Encephalitis Consortium list of co-investigators**

| Owen White | Alfred Health, Melbourne, VIC, Australia |
| --- | --- |
| Paul Beech | Alfred Health, Melbourne, VIC, Australia |
| Matt Megens | Alfred Health, Melbourne, VIC, Australia |
| Joanne Dimovitis | Austin Health, Melbourne, VIC, Australia |
| Marie O'Shea | Austin Health, Melbourne, VIC, Australia |
| Victor Zhang | Austin Health, Melbourne, VIC, Australia |
| Richard Macdonell | Austin Health, Melbourne, VIC, Australia |
| Cassie Nesbitt | Barwon Health, Geelong, VIC, Australia |
| Sharryn Savickas | Barwon Health, Geelong, VIC, Australia |
| James Broadley | Barwon Health, Geelong, VIC, Australia |
| Alison Craig | Concord Hospital, Sydney, NSW, Australia |
| Steve Reddel | Concord Hospital, Sydney, NSW, Australia |
| Sudarshini (Darshi) Ramanthan | Concord Hospital, Sydney, NSW, Australia |
| Todd Hardy | Concord Hospital, Sydney, NSW, Australia |
| Kath Buzzard | Eastern Health, Melbourne, VIC, Australia |
| Amy Brodtmann | Eastern Health, Melbourne, VIC, Australia |
| Natalie Reidy | Eastern Health, Melbourne, VIC, Australia |
| Robert Bourke | Eastern Health, Melbourne, VIC, Australia |
| Meng Tan | Gold Coast Health, QLD, Australia |
| Simon Broadley | Gold Coast Health, QLD, Australia |
| Victoria Cottam | Gold Coast Health, QLD, Australia |
| Namarata Sobarun | Gold Coast Health, QLD, Australia |
| Chris Kyndt | Melbourne Health, Melbourne, VIC, Australia |
| Dennis Velakoulis | Melbourne Health, Melbourne, VIC, Australia |
| Tomas Kalincik | Melbourne Health, Melbourne, VIC, Australia |
| WenWen Zhang | Melbourne Health, Melbourne, VIC, Australia |
| Charles Malpas | Melbourne Health, Melbourne, VIC, Australia |
| Brian Long | Monash Health, Melbourne, VIC, Australia |
| Udaya Seneviratne | Monash Health, Melbourne, VIC, Australia |
| Helmut Butzkueven | Monash University, Melbourne, VIC, Australia |
| Mastura Monif | Monash University, Melbourne, VIC, Australia |
| Nabil Seery | Monash University, Melbourne, VIC, Australia |
| Robb Wesselingh | Monash University, Melbourne, VIC, Australia |
| Sarah Griffith | Monash University, Melbourne, VIC, Australia |
| Terence O'Brien | Monash University, Melbourne, VIC, Australia |
| Tiffany Rushen | Monash University, Melbourne, VIC, Australia |
| Tracie Tan | Monash University, Melbourne, VIC, Australia |
| David Tarlinton | Monash University, Melbourne, VIC, Australia |
| Jayashri Kulkarni | Monash University, Melbourne, VIC, Australia |
| Joanne Fielding | Monash University, Melbourne, VIC, Australia |
| Meaghan Clough | Monash University, Melbourne, VIC, Australia |
| Rubina Alpitsis | Monash University, Melbourne, VIC, Australia |
| Ernie Butler | Peninsula Health, Melbourne, VIC, Australia |
| Andrew Swayne | Princess Alexandra, Brisbane, QLD, Australia |
| Genevieve Skinner | Princess Alexandra, Brisbane, QLD, Australia |
| Laurie McLaughlin | Princess Alexandra, Brisbane, QLD, Australia |
| Michael Hayes | Princess Alexandra, Brisbane, QLD, Australia |
| Nicola Warren | Princess Alexandra, Brisbane, QLD, Australia |
| Stefan Blum | Princess Alexandra, Brisbane, QLD, Australia |
| Richard Wong | QLD Pathology |
| Bruce Taylor | Royal Hobart, Hobart, TAS, Australia |
| Jenny MacIntyre | Royal Hobart, Hobart, TAS, Australia |
| Amy Halliday | St Vincent's, Melbourne, VIC, Australia |
| Wendyl D’Souza | St Vincent's, Melbourne, VIC, Australia |
| David Yang Tran | St Vincent's, Melbourne, VIC, Australia |
| Catherine Meade | St Vincent's, Melbourne, VIC, Australia |
| David Gillis | Sunshine Coast Hospital, QLD, Australia |
| Josh Barton | Sunshine Coast Hospital, QLD, Australia |
